# Measuring asymmetry from high-density 3D surface scans: An application to human faces

**DOI:** 10.1371/journal.pone.0207895

**Published:** 2018-12-26

**Authors:** Omid Ekrami, Peter Claes, Julie D. White, Arslan A. Zaidi, Mark D. Shriver, Stefan Van Dongen

**Affiliations:** 1 Evolutionary Ecology Group, Department of Biology, University of Antwerp, Antwerp, Belgium; 2 Medical Imaging Research Center (MIRC), Department of Electrical Engineering–ESAT, Faculty of Engineering, KU Leuven, Leuven, Belgium; 3 Department of Anthropology, The Pennsylvania State University, University Park, Pennsylvania, United States of America; Liverpool John Moores University, UNITED KINGDOM

## Abstract

Perfect bilateral symmetry is the optimal outcome of the development of bilateral traits in the absence of developmental perturbations. Any random perturbation in this perfect symmetrical state is called Fluctuating Asymmetry (FA). Many studies have been conducted on FA as an indicator of Developmental Instability (DI) and its possible link with stress and individual quality in general and with attractiveness, health and level of masculinity or femininity in humans. Most human studies of facial asymmetry use 2D pictures and a limited number of landmarks. We developed a protocol to utilize high-density 3D scans of human faces to measure the level of asymmetry. A completely symmetric spatially dense anthropometric mask with paired vertices is non-rigidly mapped on target faces using an Iterative Closest Point (ICP) registration algorithm. A set of 19 manually indicated landmarks were used to validate the mapping precision. The protocol’s accuracy in FA calculation is assessed, and results show that a spatially dense approach is more accurate. In addition, it generates an integrated asymmetry estimate across the entire face. Finally, the automatic nature of the protocol provides a great advantage by omitting the tedious step of manual landmark indication on the biological structure of interest.

## Introduction

While symmetry is often considered as the optimal morphological state for many bilateral traits, perfect symmetry hardly ever exists [1, 2, 3]. Causes of asymmetry can be categorized into three groups: a) Congenital or related to genes, b) developmental and c) acquired (caused by accidents or diseases) [4]. From a morphological perspective, generally, three types of asymmetry are distinguished on the basis of the distribution of asymmetries at the population level, namely Directional Asymmetry (DA), Fluctuating Asymmetry (FA) and Anti-Symmetry (AS) [5]. DA is referred to a state where one side of the body develops to be bigger than the other side and it can be defined as the difference between the population level averages of right and left sides of the body. AS occurs when DA is present in a population, but the direction varies between individuals. FA is defined as directionally random perturbations from perfect symmetry (or DA) and is believed to represent the stochastic nature of development. These three types of asymmetry are not mutually exclusive and can co-occur in the same population [6]. While DA and AS are considered to be population-level traits, FA has been studied at both the population and individual level. In theory, FA is considered to reflect the precision of development and increasing levels of FA are expected to emerge due to lower Developmental Stability–the capacity of an organism to buffer the genetic and environmental stresses that tend to affect its development [7, 8, 9]. It has therefore been studied extensively in ecology and evolutionary biology and is proposed to be an indicator of environmental and genetic stress [10,11], or an indicator of individual quality [12, 13, 14] in a variety of organisms. However, this field of research has seen an enormous heterogeneity in results leading to the conclusion that FA may reflect stress and quality in some cases, but certainly not consistently so.

A specific property of FA is that it is usually very small and difficult to measure [12]. Not only does this cause a problem in terms of repeatability and confounding of FA measures with measurement error (ME), it also makes the measurement of FA a very tedious and time-consuming exercise. The aim of this paper is to develop a methodology that provides accurate estimates using an automatic algorithm based on 3D scans. We develop the technique for human 3D face scans, but the approach is applicable to any bilaterally symmetric 3D object. In order to measure the asymmetry in human faces or bodies, different approaches have been used. Most often linear measurements on arms and legs or manual landmarking on conventional 2D photographs are applied to capture the biological structure of interest. As such, simple morphometric measurements like angles and ratios [15], or distances of specific anatomical landmarks with respect to the estimated sagittal plane [16] are the more conventional methods of calculating asymmetry. Of course, since the human face and body are 3D structures it is reasonable to assume that representing them by 2D images comes with the cost of losing potentially useful information embedded in the disregarded third dimension [17, 18]. Therefore, with the emergence of 3D scanning technology, more recent studies have focused on using 3D techniques to evaluate asymmetry[19, 20, 21]. These studies use a configuration of manually indicated landmarks, consisting of paired landmarks on each side as well as unpaired landmarks positioned on the mid-plane of the structure. This configuration is then mirrored with respect to an arbitrary plane (in most cases the x = 0 plane) and the landmarks are relabeled to regain their correspondence with the original configuration. By performing a least-squares rigid transformation (Procrustes alignment) the two set of landmarks are super-imposed and the asymmetry value is calculated by simply subtracting the mirrored configuration from the original. So far, this method has been widely accepted by scientists as a well-established method of FA calculation. However, the magnitude of FA distribution is small and sometimes within the range of measurement error [22]. Therefore, the human error introduced by manually indicating the landmarks can cause some inaccuracy in the calculation of FA values. Also due to the sparse nature of the landmark configuration, there is a possibility of losing some dominant features of the face or the body **[23]**. Some other studies have taken an automatic surface-based approach on analyzing asymmetry, using commercially available software packages [24, 25, 26]. In these studies, the asymmetry is obtained as the distance between the two surfaces of a face and its mirror with respect to the sagittal plane. A shortcoming of this approach is that it only measures the component of asymmetry that is perpendicular to the surface of the face. This can introduce significant error, especially in case of FA where the magnitude of asymmetry is usually small. Also in such methods there is no possibility for separating FA and DA from the measured asymmetry in a standardized way for a population.

We overcome these difficulties by extending the method described above to an automated spatially dense protocol on asymmetry assessment. First, we describe the construction of our paired symmetric anthropometric mask, based on the mask used by Claes et al. (2011). The original mask consisted of points that were sampled in a non-paired and non-symmetric manner, meaning the points on either side did *not* have an exact replicate on the other side. Having a symmetrical paired mask enables us to obtain mirrors of the faces by just reflecting and relabeling the landmarks **[17, 19]**. We can also use this mask to simulate asymmetric faces with known amounts of asymmetry for each vertex. This way we can validate the ability of our algorithm to localize asymmetry in faces. We then describe the different steps of our mapping algorithm used to transfer the faces into a homogenous configuration by non-rigidly mapping our mask onto each face. Next, we evaluate our mapping algorithm by comparing 19 automatically obtained anatomically significant landmarks (around the nose, eyes and the lips) with their manually indicated counterparts. We then perform a simulation study to illustrate the performance and benefit of our spatially dense and automated approach in calculating FA over the traditional use of sparse landmarks. Also, the algorithm’s ability in localizing FA is tested and discussed.

## Materials & methods

**Fig 1:**
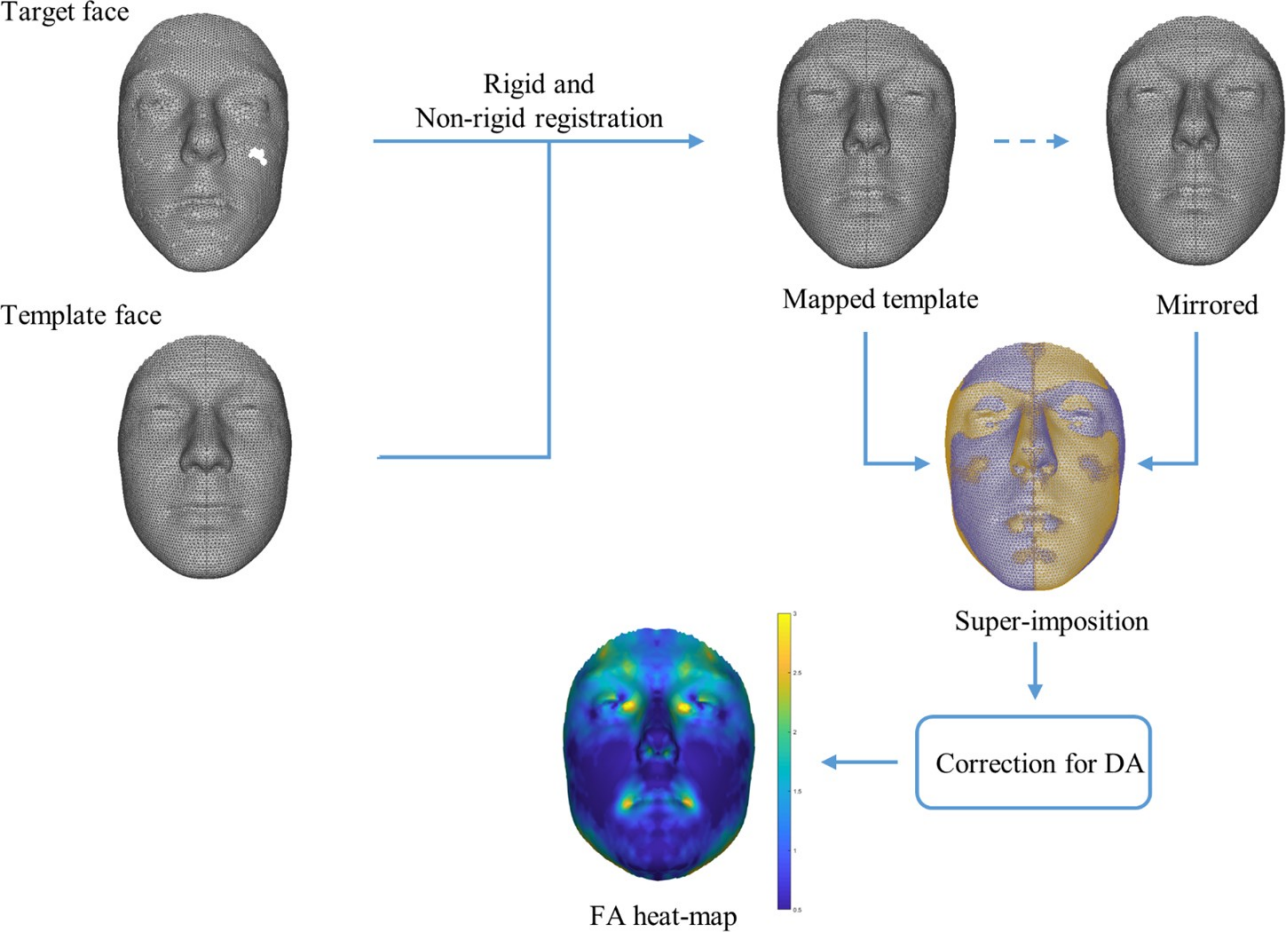
Complete flow diagram of the proposed spatially dense approach for FA calculation.

Objects in 3D computer graphics are represented using a collection of *vertices* (coordinates of points in 3D) and *edges* (the connections between the vertices). The output surfaces of 3D scanners may have a different number of vertices, different edge configuration, and in some cases, parts of the surface of interest might be missing. In order to be able to use a standardized and homologous asymmetry assessment method on all of the faces, first these faces should all be represented by the same configuration of vertices and edges. This is achieved through mapping a paired symmetrical spatially-dense anthropometric mask onto each one of the faces in our dataset. Finding the mapping function between two surfaces is called 3D surface registration. Fig 1 shows the complete flow diagram of the method, displaying the steps starting from the target and template face and leading to the calculated FA heat-map.

### Anthropometric mask

We used the mask developed by Claes (2012) as a base to obtain our paired symmetrical mask. The original mask covers the facial area of interest, consisting of spatially-dense, uniformly sampled (equally distanced at ~2 mm) points on an averaged facial form calculated over 400 Western Australian healthy young individuals between the ages of 18–25 years [27].

We cut the mask in the middle into two hemi-faces at the *x* = 0 plane, which due to explicit construction resulting from participants sitting upright in their natural head position during scanning, it roughly equates the sagittal plane. The right hemi-face was then mirrored with respect to the same plane. The original hemi-face and its mirrored version were then stitched together by creating a *mid-line*. This was done by merging corresponding vertices that were located close to each other (the threshold was arbitrarily chosen as 0.3 of the average edge length) into new vertices on the plane *x* = 0. The outcome facial mesh was then “cleaned” at the mid-line by *collapsing* or *splitting* the edges based on their length and removing any duplicate or unreferenced vertices. This resulted in a symmetrical facial mask consisting of 7160 quasi-landmarks, with 3527 paired quasi-landmarks on each side and 106 unpaired quasi-landmarks on the mid-line of the face.

### Mapping

The mapping process is comparable to fitting an elastic mask onto a solid face **[17].** Based on the Iterative Closest Point (ICP) method **[28]** and the work of [29, 30, 31], the algorithm maps the *template* mask *M* onto the *target* face *T* by non-rigidly deforming *M* according to anatomical and geometrical features of *T*.

The different steps of the algorithm are presented in the pseudo-code below:

**Start**

1. **Rough alignment**
  - **Calculate rigid transformation matrix *T***_***mat***_ **based on 5 positioning landmarks on *M* and *T***
  - **Apply *T***_***mat***_ **on *T* to roughly align it with *M***
2. **Rigid ICP**
  - **Select a number of sample vertices from *M* and *T* using a stable sampling method**
  - **Start iteration by finding two-way fuzzy correspondence between sampled vertices on *M* and *T***
  - **Correct for outliers based on distance and the overlapping area of the meshes**
  - **Calculate the transfer matrix and apply to *T***
  - **Re-iterate until convergence**
3. **Non-rigid registration**
  - **Find two-way fuzzy correspondence between vertices p_m_ on *M* and vertices q_t_ on *T* using *K-NN***
  - **Correct for outliers (Distance-based, Normal-based, Holes)**
  - **Calculate displacement matrix *F***_***M***_
  - **Convolve *F*_*M*_ with a Gaussian with variance σ^2^_1_ (Viscous behavior)**
  - **Update global displacement field *D***_***g***_ **by adding the regularized *F***_***M***_
  - **Convolve *D*_*g*_ with a Gaussian with variance σ^2^_2_ (Elastic behavior)**
  - **Update *M* by adding *D***_***g***_
  - **Decrease *k* and *ρ***
  - **Re-iterate until convergence**

**End**

**Step 1- Rough alignment:** We start by using 5 roughly indicated landmarks on *M* and *T* (the outer corners of the eyes, the tip of the nose and the corners of the mouth) to crudely align the surfaces using a superimposition of the points (scaling, rotation, and translation). This superimposition is done by finding the transformation matrix using a least-squares fitting of the two sets of landmarks [32] and applying the transformation to the whole face.

**Step 2- Rigid ICP:** In this step, we rigidly align the two surfaces as closely as possible. The ICP method introduced by Besl & McKay (1992) is probably the most widely used algorithm for rigid registration of point-clouds. Different survey studies on rigid registration methods show ICP to be faster and more accurate compared to other common methods, especially in cases in which the two point-sets have a good rough alignment [33, 34, 35]. We start by sampling each of the faces using a stable sampling method **[36].** This method selects the samples that constrain potentially unstable transformations. Unstable transformations occur when the sampled points come from feature-less regions and the surfaces can *slide* over each other without a significant change in the error metric (e.g. aligning two planes). This method tries to minimize the instability by drawing a set of sample points primarily from stable, i.e. “lock and key”, areas of the input meshes. In other words, the algorithm favors samples from non-flat areas of the meshes. Then iterations start by establishing two-way correspondences between the sampled vertices of both surfaces. The found correspondences are then corrected for outliers by checking the distances between the corresponding vertices. Corresponding vertices with distances more than one standard deviation away from the mean value are neglected. We also checked for correspondences at the boundaries of the two surfaces (as explained in step 4), in order to make sure that the correspondences are limited to the area of overlap between the faces. The best rigid transformation that maps the sampled vertices on *T* to the sampled vertices on *M* is computed using least-squares fitting and applied to *T*. The algorithm continues until the average change in *T* falls below 0.0001 mm or the number of iterations reaches 100.

**Step 3- Non-rigid registration:** In all cases, *T* has a significant morphological difference compared to *M*. Therefore, a rigid registration will not suffice to completely map the two surfaces. In this step we follow an approach similar to the previous step, however this time the transformation model allows shape changes in *M* (hence the term non-rigid). Due to the aforementioned morphological difference, simply using a one-way registration will not be able to properly capture the structure of *T* in all cases. To overcome this problem, we opt for *symmetrical correspondence*. This means we look for correspondences for both *M* and *T*. By using symmetric correspondences, we create pull forces from the target, in addition to the usual push forces from the mask **[37].** This can help to capture any possible out-of-reach vertices on the target, therefore any possible protrusions on the target surface will not be neglected **[38].** After finding the two-way correspondences, they are merged into one matrix and used to obtain the deformation field for *M*.

The non-rigid registration step starts by finding correspondences for vertices *P* = {p_1_,…,p_m_} on *M*, chosen from vertices *Q* = {q_t_,…,q_t_} on *T*, and vice versa. We used Distance-Weighted K-Nearest Neighbors (KNN) method [39, 35] to establish such dense correspondences. The basic idea here is to relax the binary correspondence between the two surfaces to form a continuous correspondence matrix **[40]**. In this way, instead of a one-on-one correspondence between the mask and target vertices, each vertex p_*m*_ gets assigned to a number (*k*) of vertices q_t_ on the target, for which the correspondence is weighted based on their distance.

To correct for possible outliers, the found correspondences are refined by comparing the surface normals and the Euclidean distance between the corresponding points, as well as the possibility of presence of holes on *T*. If there is a relatively high distance between the corresponding vertices or a high angle between their respective normal vectors, the correspondence is set as an outlier. The threshold for distance is defined as three standard deviations away from the average distance between the corresponding points. The threshold for normal angle difference was set empirically as *π*/12 to avoid correspondences between points that belong to the regions that are anatomically different. The correction for holes is done by checking the found correspondences for any vertices on the boundary edges of *T*. These are the vertices that the edges (connections) between them are only shared by one triangle on the mesh. If any of these 3 groups of outlier vertices are present in the correspondences, the respective correspondence weight is set to zero and their displacement is only set by their neighbors.

The correspondences for each surface are obtained by calculating the weighted average of the corresponding vertices on the other surface. This results in new sets of corresponding vertices *S* = {s_1_,…,s_m_} and *R* = {r_1_,…,r_t_}, for *P* and *Q* respectively. Then the displacement matrix *F*_*M*_ is defined as:

$$F_{M}=\left[\begin{array}{c} S\\ Q \end{array}\right]-\left[\begin{array}{c} P\\ R \end{array}\right]$$ (1)

For this step, we used a Visco-Elastic regularization based on Motion Coherence Theory by Yuille & Grzywacz (1989) and Bro-Nielsen (1997). This theory suggests that points that are located close to each other should move coherently, therefore the velocity field (in our case the displacement matrix) should be smoothened and regularized based on a defined energy function [41, 42]. In a benchmark study with an application similar to ours, Snyders (2014) concludes that using a Visco-Elastic regularization will result in the most accurate mapping amongst the studied methods [38]. As the name suggests, this method uses a physical model to regularize the deformations. The algorithm works as below:

In order to maintain the smoothness of the surfaces, the obtained displacement matrix *F*_*M*_ is convolved with a 3D Gaussian kernel with variance *σ*^2^_1_ decreasing from 30 to 1 (mm^2^) linearly at each iteration. By doing so, the displacement of every point will have an effect on its neighboring points. In other words, as a point moves towards its corresponding point, it drags its neighbors along. This ensures that the points that lie in a neighborhood will move coherently and the surface will remain smooth, i.e. the surface shows a viscous behavior. The smoothened displacement matrix is then added to the global displacement field D_g_ which is updated at each iteration. D_g_ is also smoothened by convolving with the same Gaussian. This adds the elastic characteristic to the surface which ensures that the surface stays homologous to its original shape. Surface *M* is then updated by adding the regularized global displacement field. The process is re-iterated until the error value reaches below 0.001 mm or the number of iterations exceeds 150.

We also applied the concept of deterministic annealing **[43]** in our registration algorithm. In this method, the number *K* of corresponding vertices for each vertex decreases iteratively form 25 to 5. Also, the radius of effect of the applied Gaussian is decreased at each iteration, by changing variance *σ*^2^_2_ from 3 to 1 (mm^2^). This causes the search for correspondence and the following deformations to start more globally during the initial iterations and move towards more localized deformations at each iteration. This helps the algorithm to avoid falling into a local minima as well as increasing the computational efficiency.

By using the above-described method to map the mask face on all of our target faces, the surface outputs of a 3D scanner are now represented with a standardized and homogenous configuration.

### Parameter sweep

In each of the sections described above, a number of parameters were used as part of the algorithm. These parameters are listed below:

- ***The number of sampled vertices in step 2***
- ***The number of found correspondences*, *k*, *in step 3***
- ***Variance of Gaussian1*, *σ***^**2**^_**1**_, ***in step 3***
- ***Variance of Gaussian2*, *σ***^**2**^_**2**_, ***in step 3***
- ***Distance-based and normal-based outlier thresholds*, *in step 3***
- ***Convergence thresholds*, *in steps 2 and 3***

The best configuration for the value or the range of these parameters were set by performing a “*parameter sweep*”. This means that the value of one parameter is increased or decreased step by step while all the other parameters are kept constant. The average error, as described in *mapping validation* section, is calculated for each step and the parameter is fixed on the value corresponding to the lowest error. We then move to the next parameters one by one until all of the parameters are set to their optimal value or range. The results of the parameter sweep are added as Supporting Information (S1–S12 Figs).

### Mirroring and super-imposition

Since we used a template mask with paired landmarks, an approach similar to the method proposed by [19] can be taken here to obtain the mirrored version of the faces. This is done by reversing the signs of the x-coordinate of each vertex, then relabeling the paired vertices with their counterpart in order to re-establish the compatibility of the original face and the mirrored version. The symmetric consensus -or the average face- is then obtained by simply averaging over all of the original faces and their mirrors. We did this by using a General Procrustes Analysis, in which the original faces and their mirrors are iteratively superimposed onto the symmetric consensus and the consensus is updated in each iteration until the algorithm converges. To have a more accurate estimation of asymmetry, the superimposition should be carried out based on the symmetric parts of the face. For this, we applied the robust least-squares superimposition method [17]. In this method, a statistically meaningful approach is taken to estimate the points lying on the asymmetric regions of the face (outliers) and relevant weights are assigned to each point. The faces are then superimposed onto the symmetric consensus in a weighted least-squares manner.

### FA and DA calculation

The asymmetry of the face can be obtained by calculating the difference between the original and mirrored faces after the superimposition step, or equivalently comparing them with the average of both. The obtained values constitute a combination of FA as well as DA of the face. As explained before, DA can be quantified as the signed difference between the average of all the faces in a population and the average of their reflections. We write this as:

$$\frac{\sum_{i=1}^{n}\left(O_{i}-R_{i}\right)}{n}$$ (2)

where *O*_*i*_ represents the original configuration of face *i* and R_i_ represents the reflected version. By subtracting DA from the vertex-specific signed asymmetries, FA of face *i* is obtained (2).

$${FA}_{i}=|(O_{i}-R_{i})-\frac{\sum_{i=1}^{n}\left(O_{i}-R_{i}\right)}{n}|$$ (3)

### Mapping validation

**Fig 2:**
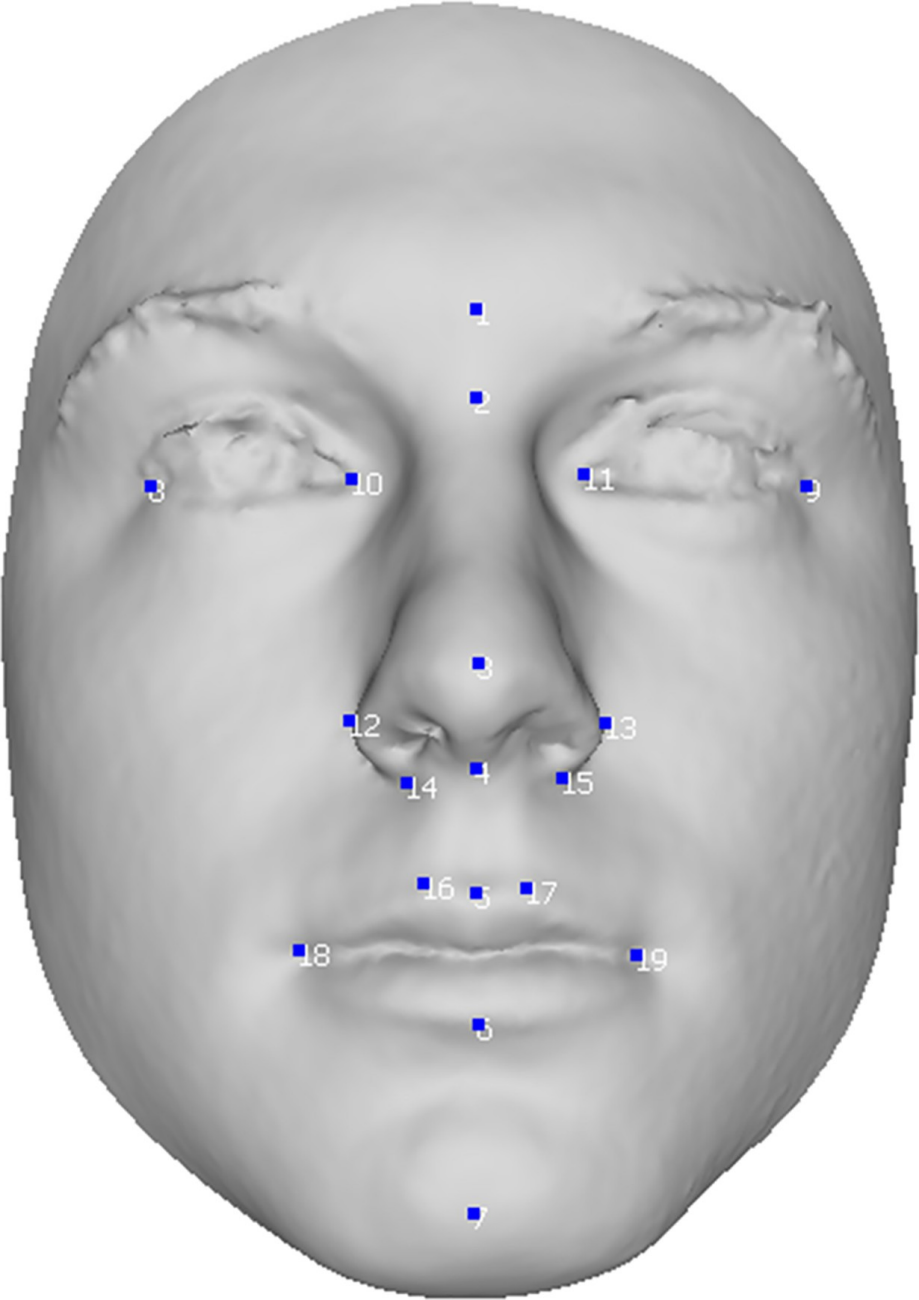
The position of the 19 manual landmarks used for mapping validation.

In order to validate the precision of our mapping algorithm, we used a dataset of 30 facial scans of adult females and males between 18 and 30 years old, with different levels of asymmetry and mixed self-reported ancestries. The scans have been captured at various locations over years by the Department of Anthropology, Pennsylvania state university, using 3dMDface 2-pod and 3-pod system, of which the precision and repeatability are previously tested and validated to be sub-millimeter **[44]**. The Pennsylvania State University Institutional review board (IRB) approved the collection of data from the participants recruited at the following locations: State College, PA (IRB 44929 and 4320); New York, NY (IRB 45727); Urbana-Champaign, IL (IRB 13103); Dublin, Ireland; Rome, Italy; Warsaw, Poland; and Porto, Portugal (IRB 32341). All participants signed a written consent form before participation. A set of manually indicated landmarks, consisting of 19 anatomically significant points on the face (7 non-paired, 12 paired) was used as a basis for mapping validation (Fig 2). The set of manual landmarks were indicated by 2 different users (P1 and P2), who both repeated the indication 3 independent times.

**Fig 3:**
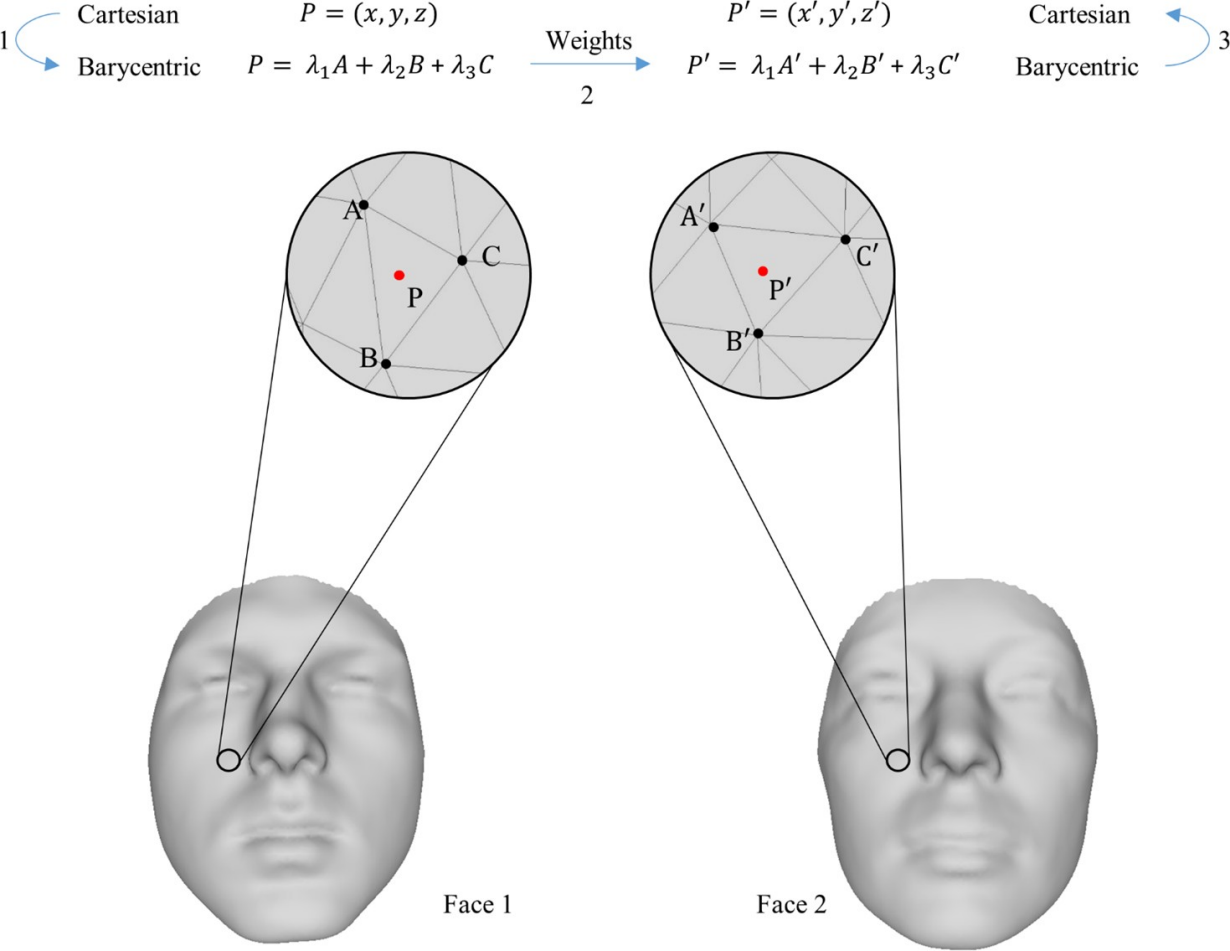
Using barycentric coordinates to transfer a landmark from one face to another. The barycentric coordinates of point P are calculated with respect to triangle ABC. The obtained weights are then used in combination with the Euclidean coordinates of triangle A′B′C′ in Face2 to obtain the coordinates of P′.

We used the manual landmark indications to automatically obtain the corresponding set of anatomical landmarks on the template mask. For each face, the average of all of its manually indicated landmarks was obtained and the barycentric coordinates of these average landmarks were calculated with respect to the mapped template [45]. In many cases these average landmarks were not exactly located on the surface, accordingly, their projection on the nearest face was used instead. Barycentric coordinates define any point inside a triangle by assigning weights to each of the triangle vertices (corners) in a manner that the weighted average of the vertices equals the coordinates of that specific point. To find the coordinates of landmark *P′* on Face2 which corresponds to landmark *P* on Face1, we first calculate the barycentric coordinates of *P* with respect to its surrounding triangle *ABC* on Face1. The calculated barycentric weights are then transferred to triangle *A′B′C′* on Face2 which structurally corresponds to triangle *ABC* on Face1, but with different Cartesian coordinates due to the difference in the shape of the faces. In a sense, by using barycentric coordinates, we are in fact “stitching” a landmark to the template and transferring it to another face. The Euclidean coordinates of the corresponding point *P′* are then calculated using the barycentric weights and the coordinates of *A′B′C′* (Fig 3). By utilizing this technique in a leave-one-out strategy, the automatically obtained landmarks for an individual face were calculated as the average of the transferred barycentric coordinates of (n-1) set of landmarks from all of the other faces. The automatically obtained landmarks were compared with their corresponding manual indications by means of Euclidean distance.

The symmetric faces, obtained by averaging each of the original faces and their reflected instances, were used to validate the FA calculations. Known values of random asymmetry were added to the symmetric faces. This was done by choosing a random sparse subset of 100 vertices on each face and adding random displacements with normally distributed magnitudes to the subset. To maintain the smoothness of the surface, Radial Basis Functions (RBFs) with small radiuses were applied **[31].** For each face, 10 different levels (magnitudes) of asymmetry were added, resulting in a total of 300 faces. We then mapped our template face onto these simulated faces using the developed registration algorithm. The remapped simulated asymmetric faces were then given as input to our FA calculator algorithm and the results were obtained. We also tested our algorithm’s ability in localizing the asymmetric regions using the simulated asymmetric faces. The correlation between the actual asymmetry value of each vertex and the calculated asymmetry using our algorithm was examined.

## Results

**Fig 4:**
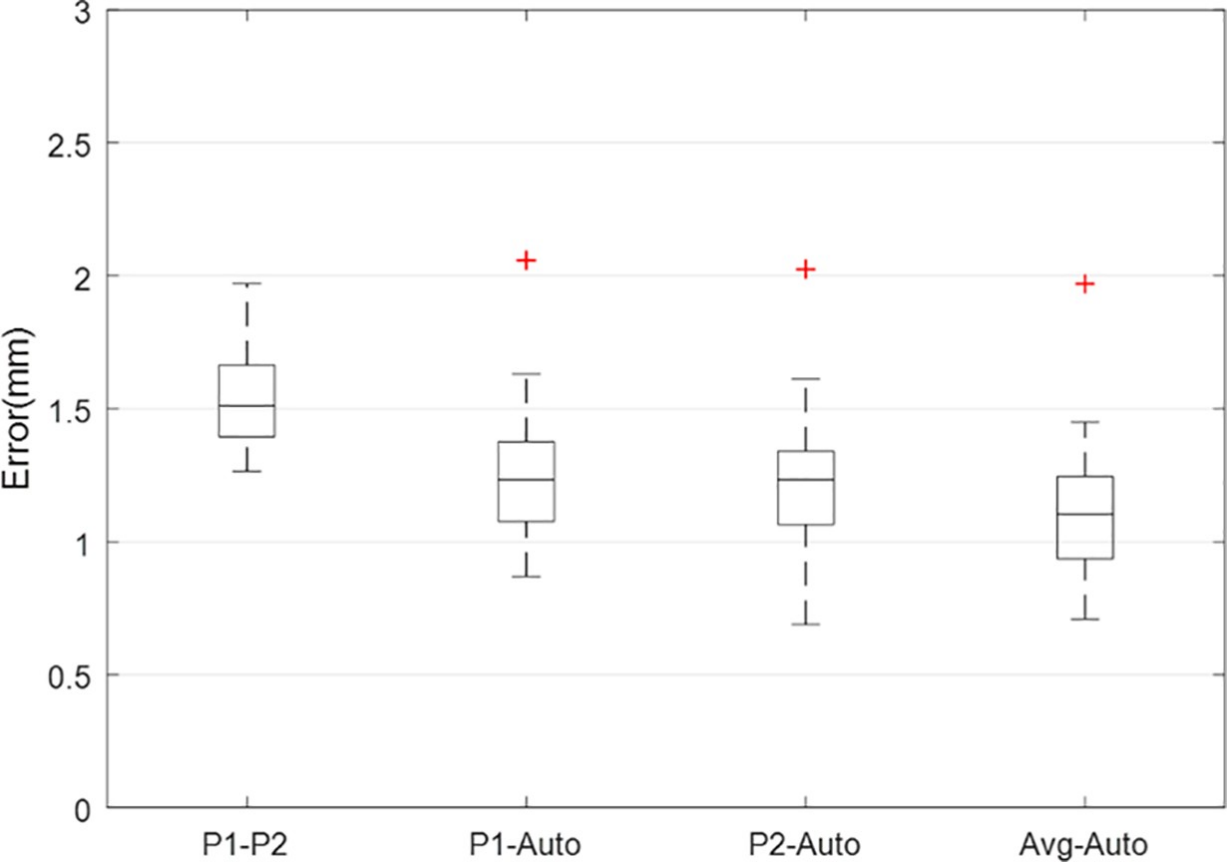
Boxplot chart of average landmark error between manual indications and automatic landmarks. The plot shows the error between the average of manual indications of user 1 and user 2 (P1-P2), the error between the average of manual indications of user 1 and the algorithm (P1-Auto), the error between the average of manual indications of user 2 and the algorithm (P1-Auto), and the error between the average of manual indications of both users and the algorithm (Avg-Auto).

The remapping process of each face took about 2 minutes on a computer with core i7 dual-core 2.7 GHz processors and 16GB of RAM. The average error for each face was calculated as the RMSE of all its 19 landmarks. Fig 4 shows the results of our mapping validation test. The boxplot displays the distribution of the average landmark error values between the user indications (P1-P2), between the users and the automatic landmarks extracted from the mapped template mask (P1-Auto and P2-Auto) and between the average of all sets (2 times 3) of manual landmarks and the automatic landmarks (Avg-Auto).

**Fig 5:**
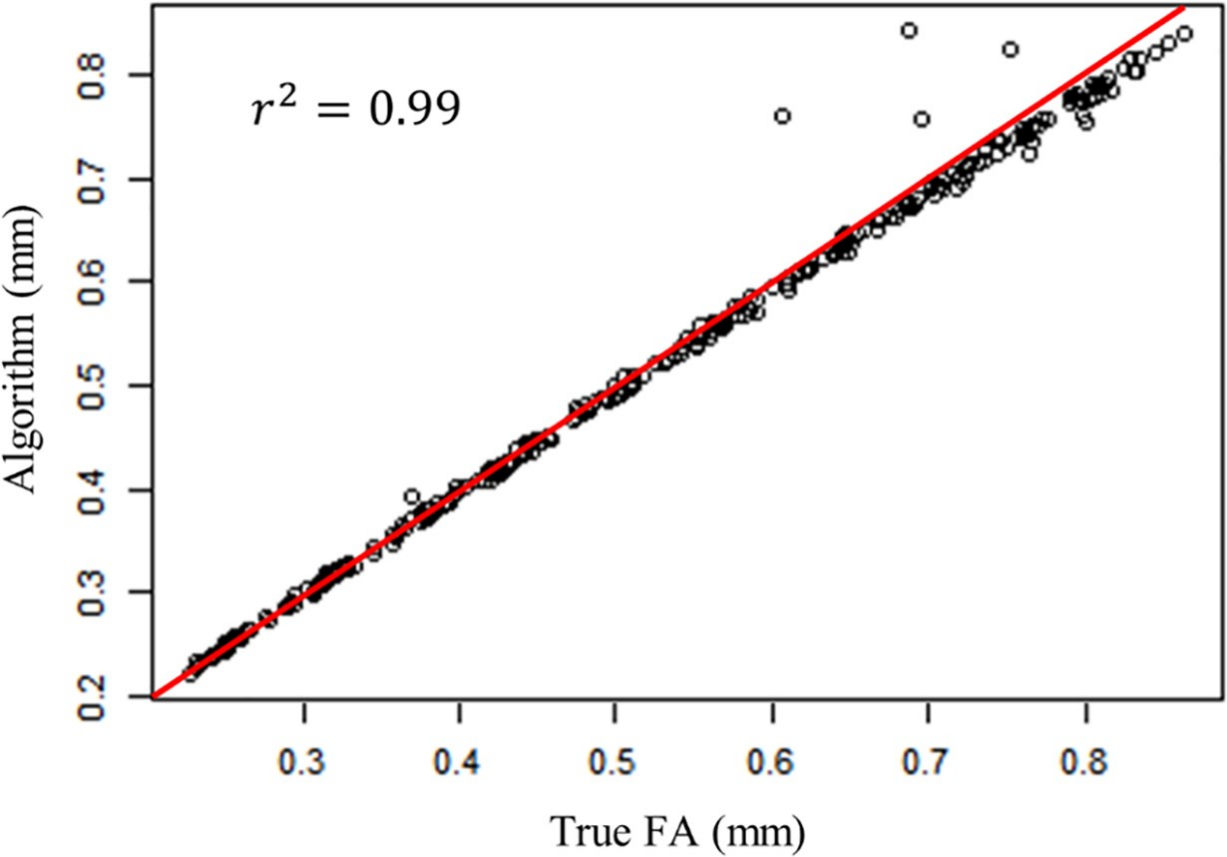
Comparison between true simulated FA values (True FA), the FA values using all vertices (Algorithm).

The 300 simulated faces with different levels of asymmetry, as described in *mapping validation* section, were used to assess our algorithm’s ability in capturing the average facial asymmetry. Fig 5 shows the comparison between the true average FA values based on all points (True FA), average FA values calculated by our algorithm based on all points (Algorithm).

**Fig 6:**
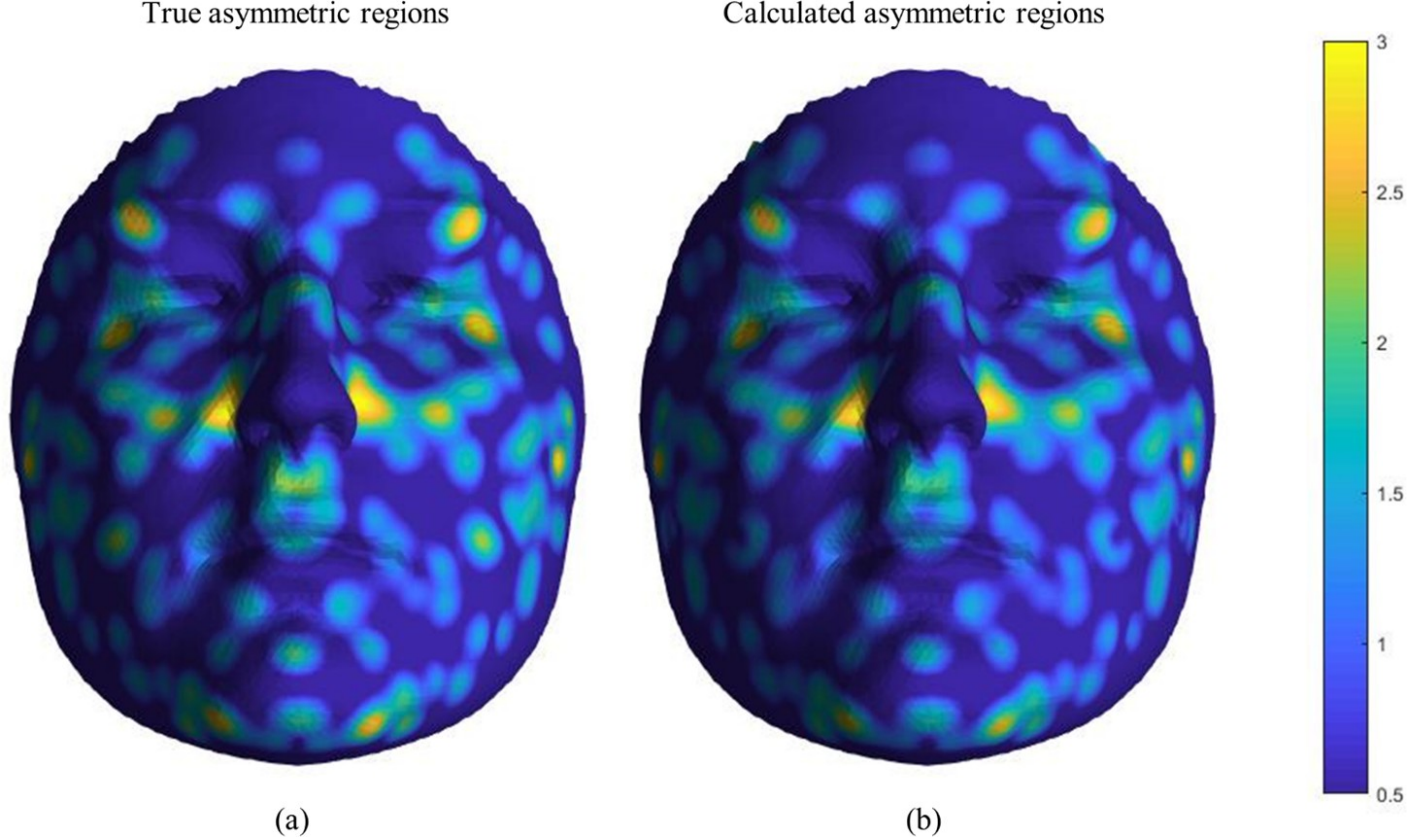
Asymmetry heat-map of a sample simulated face. (a) and (b) show the true and the calculated respectively.

**Fig 7:**
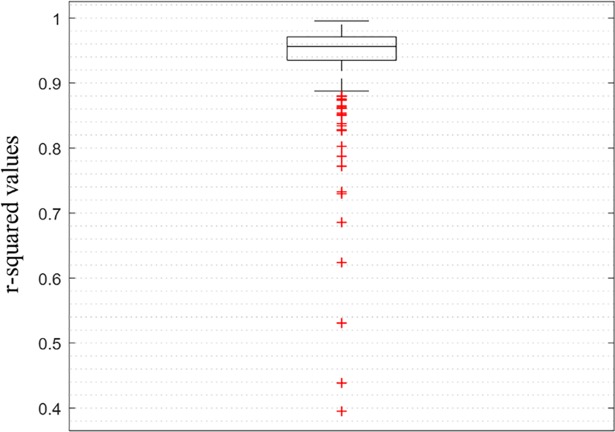
Box-plot of the R^2^ values of all 300 simulated faces for the precision of the algorithm in localizing the FA values.

We also used the simulated faces to evaluate our algorithm’s ability in localizing asymmetry. Fig 6 shows the true asymmetry heat-map of one of the simulated faces with high amounts of FA (a) and the calculated asymmetry heat-map (b). The correlation between the true and calculated FA values for all of the vertices of a face was calculated for all 300 of the simulated faces and the R-squared values are represented by the box-plot in Fig 7.

## Discussion

In this study, we developed an algorithm to analyze and quantify asymmetry, especially FA, in 3D scans of human faces by taking a spatially dense approach. Conventional methods of asymmetry analysis mostly use a limited number of landmarks to represent the structure of interest in normal 2D photographs. This can lead to an inaccurate estimation of the asymmetry present in the structure, especially in complex structures such as human faces which can show various amounts of asymmetry in different regions. Representing a 3D structure with 2D images can also result in wrong calculations of asymmetry due to the loss of information by discarding one dimension. Also, the landmarks have to be manually selected on the structure, which can be highly time-consuming and lack precision and repeatability. A few studies that have used 3D scans to calculate asymmetry also used a number of manually assigned landmarks for their purpose or have only measured the component of asymmetry perpendicular to the surface. We overcame these problems by taking an automatic and spatially dense approach. First, the scans needed to be transferred into a standardized and homogeneous setting, where all the faces are represented using the same setting of vertices and edges. This was done by non-rigidly registering an average template face onto each of the faces in our dataset. The template face used for this study was produced to have paired landmarks on each side and non-paired landmarks on the mid-line. Using a template with paired vertices enables us to obtain the reflection of a face by just inverting the vertices with respect to the midline and relabeling them, whereas in Claes et al. (2011) an additional step of mapping the template on the mirrored face was needed. This significantly reduced the computation time and avoids additional errors introduced by the second mapping required previously. Using a paired template also enabled us to simulate asymmetric faces with precisely known amounts of added asymmetry which was used to validate our FA calculation algorithm.

We developed a non-rigid registration algorithm based on the work of Yuille & Gryzwacz (1989) and Bro-Nielsen (1997), where the template face was iteratively mapped onto the target faces. We then proceeded to develop an algorithm to estimate the asymmetry of the remapped faces. This was done by superimposing the original and reflected faces using a generalized Procrustes analysis and calculating the difference between each face and its mirrored version. The asymmetry was then calculated and corrected for possible DA value.

Mapping validation (Fig 4) showed that the average error was low (below 1.3mm) and somewhat smaller than the error associated with the manual indications. Thus, our algorithm can detect the anatomy of the face accurately and presents a suitable alternative to manual landmarking in terms of measurement error. However, it should be noted that the validation results are only based on 19 landmarks, and validating the mapping for the entire face is practically impossible. Nevertheless, these 19 landmarks cover the anatomically significant regions of the face and the rest of the regions consist of mostly ‘flat’ areas which are usually easier to map. So it is safe to assume that the algorithm shows a similar precision in performance for the entire face.

A simulation study of the performance of our approach in detecting morphological asymmetries yielded average asymmetries very close to the simulated asymmetries (Fig 5), while using conventional methods that measure the asymmetry based on a limited number of landmarks will definitely result in a weaker correlation. This difference is simply due to the spatially-dense feature of our algorithm compared to the sparse nature of other methods, which will result in missing the asymmetry present in some areas of the face. Having a spatially dense approach can help us overcome this problem by taking into account all of the surface of interest. Also, the use of a dense mask can result in a more accurate super-imposition of a face and its mirrored version when calculating FA. This difference in approaches can be more crucial in the case of small local asymmetries. Following [17], Hill et al. (2017) have also shown in their study that results of conventional 2D approaches differ significantly from 3D dense approaches [46].

Additionally, taking an automated spatially dense approach will provide us with the opportunity to analyze the asymmetry of a morphological structure in a multivariate manner. Using methods such as Principal Components Analysis (PCA) or discriminant analysis allows us to further investigate the different components and dimensionalities of asymmetry and take a step forward towards understanding the underlying processes of asymmetry. The spatially dense 3D approach also allows to separate asymmetry in the three dimensions (horizontal, vertical and depth) to evaluate their relative importance in determining overall asymmetry and their relationships with, for example, facial attractiveness (S13 Fig).

To test the ability of our algorithm in localizing FA, the true values of asymmetry in the simulated faces were compared with the values calculated by the algorithm on a vertex basis (Figs 6 and 7). We can see that overall the algorithm performs remarkably in localizing and calculating the amount of FA, although in a few cases an over-estimation of the asymmetry is visible (Fig 5). Our inspections showed that these over-estimations are caused by mapping errors mostly happening at the boundaries of the faces. This error is due to the fact that during the registration process, the found correspondences on the edges of the mesh are counted as outliers (as part of the hole detection algorithm). Consequently, the vertices located on the boundaries of the face are only affected by the push/pull forces exerted by their neighbors from one side and not all directions. Therefore, in some cases, this will lead to mapping error at the border areas. Nevertheless, the estimated average asymmetries of such faces do seem to be relevant (Fig 5). It is interesting to note here that we simulated asymmetry randomly across the face leading to a patchy distribution. In reality, facial asymmetry usually emerges in a more structural way, over larger surface areas of the face. However, this does not invalidate our approach as the algorithm is more likely to capture shape variation over larger surfaces areas more accurately compared to very patchy patterns. It can therefore be expected that more natural patterns of asymmetry will be measured even more accurately.

As we have shown in Fig 4, our algorithm has a mapping accuracy of 1.3 mm and any value below this threshold will be considered as noise. Therefore, it should also be noted that the algorithm is unable to detect any asymmetries below the mapping error. This was considered in our simulated faces, meaning the magnitudes of the added asymmetries were above the algorithm’s noise level. Moreover, the simulated asymmetries might differ from natural appearances of FA in real faces. However, the purpose of this part of the study was to evaluate the strength of our algorithm in localizing asymmetry, therefore the distribution or patterns of FA should not affect the outcome and we believe that the algorithm would perform the same when applied on real faces.

In conclusion, we clearly showed the advantages of using an automated high-density asymmetry analysis over the more conventional methods. More precisely, the spatially-dense nature of our algorithm obviously captures asymmetry more accurately compared to a sparse set of landmarks, as well as providing information on locality of the asymmetry. Through validation tests, our method was proven to be of high accuracy, both in mapping the template on the target faces and when calculating average FA. This method can also be of great benefit in studies of a high number of individuals, since the time-consuming step of manually selecting the landmarks is avoided. Also by removing the manual landmark selection step, we eliminate the introduced user error from the asymmetry calculations. Although the algorithm did show some error in localizing FA at the level of individual vertices in few of the cases, it does measure very localized asymmetry accurately and can be expected to do even better if the asymmetry occurs over larger surface areas of the face. It is thus a useful tool in studies of asymmetry. The developed algorithm can especially be advantageous in more complex structures which cannot be fully represented using a limited number of landmarks, such as body scans or skulls.

## Supporting information

S1 Fig Change of average landmark error with convergence threshold in rigid registration step. (TIF)

S2 Fig Change of average landmark error with number of samples in rigid registration step. (TIF)

S3 Fig Change of average landmark error with maximum number of correspondence in non-rigid registration step. (TIF)

S4 Fig Change of average landmark error with minimum number of correspondence in non-rigid registration. (TIF)

S5 Fig Change of average landmark error with maximum value of variance1 in non-rigid registration step. (TIF)

S6 Fig Change of average landmark error with minimum value of variance1 in non-rigid registration step. (TIF)

S7 Fig Change of average landmark error with maximum value of variance2 in non-rigid registration step. (TIF)

S8 Fig Change of average landmark error with minimum value of variance2 in non-rigid registration step. (TIF)

S9 Fig Change of average landmark error with average error threshold in non-rigid registration step. (TIF)

S10 Fig Change of average landmark error with maximum number of iterations in non-rigid registration step. (TIF)

S11 Fig Change of average landmark error with distance-based threshold for outlier correspondences. (TIF)

S12 Fig Change of average landmark error with outlier threshold based on normal angles. (TIF)

S13 Fig The separate heat-map of asymmetry in sample face from Fig 6 for 3 dimensions (horizontal, vertical and depth). (TIF)
