# Supplementary figures and images for: Measuring asymmetry from high-density 3D surface scans: An application to human faces
Source: PLoS One. 2018 Dec 26;13(12):e0207895. doi: 10.1371/journal.pone.0207895 (PMC6306226; doi:10.1371/journal.pone.0207895)

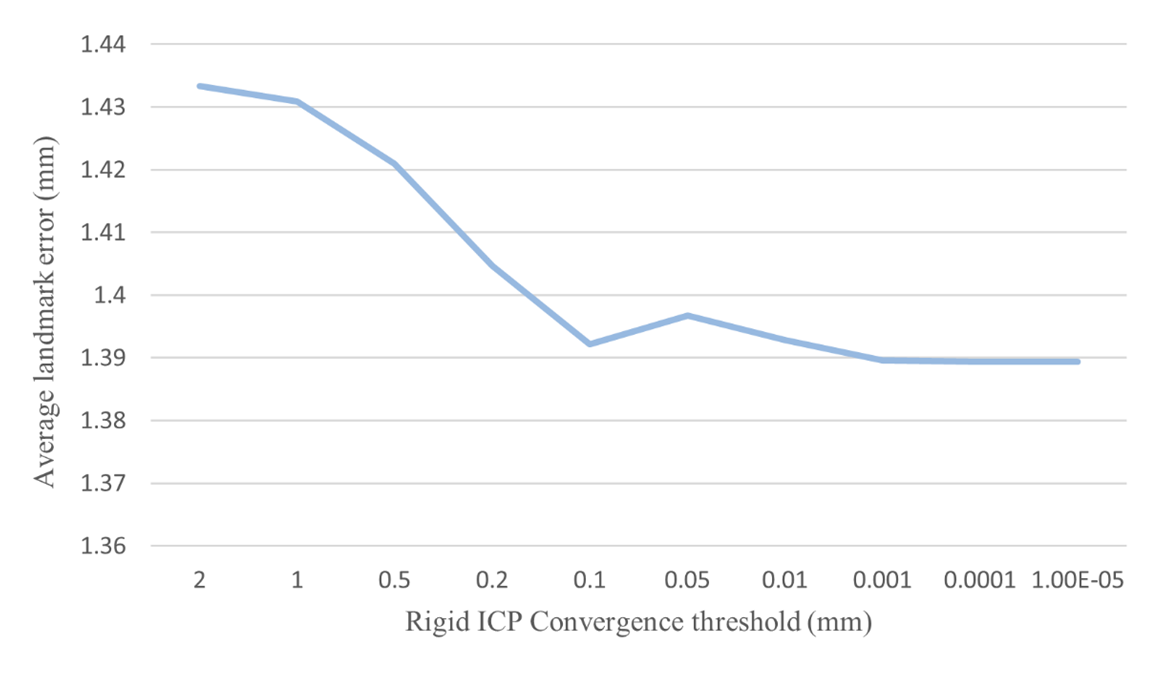

Supplement: S1 Fig — (TIF) [file pone.0207895.s001.tif]

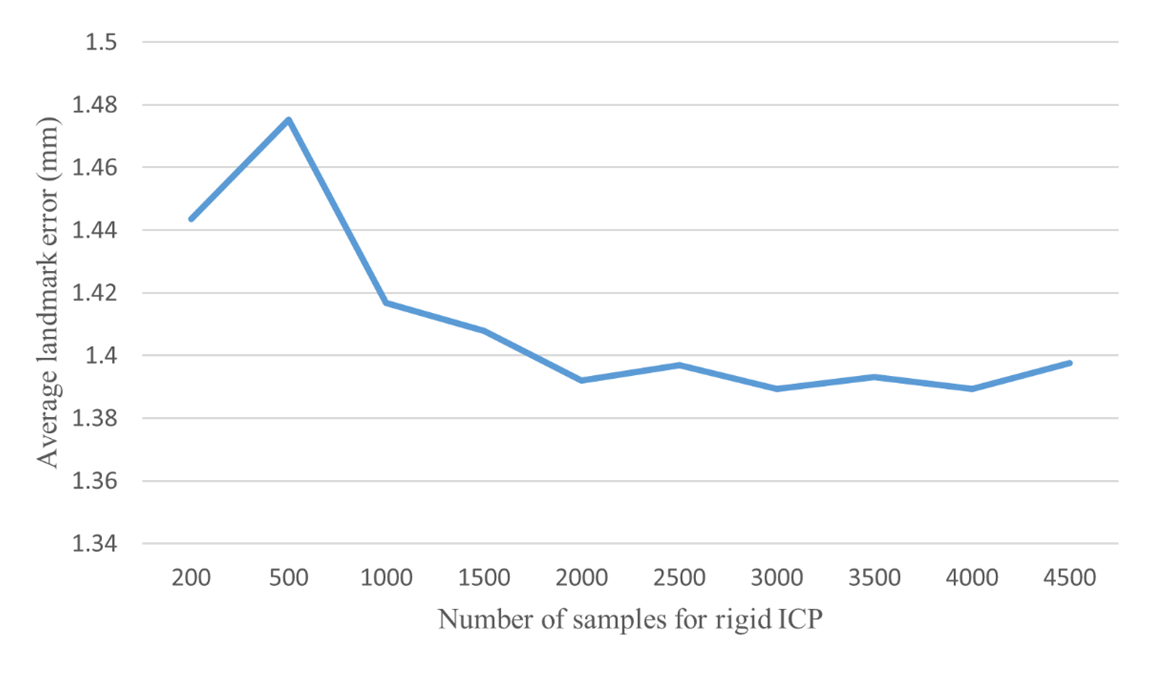

Supplement: S2 Fig — (TIF) [file pone.0207895.s002.tif]

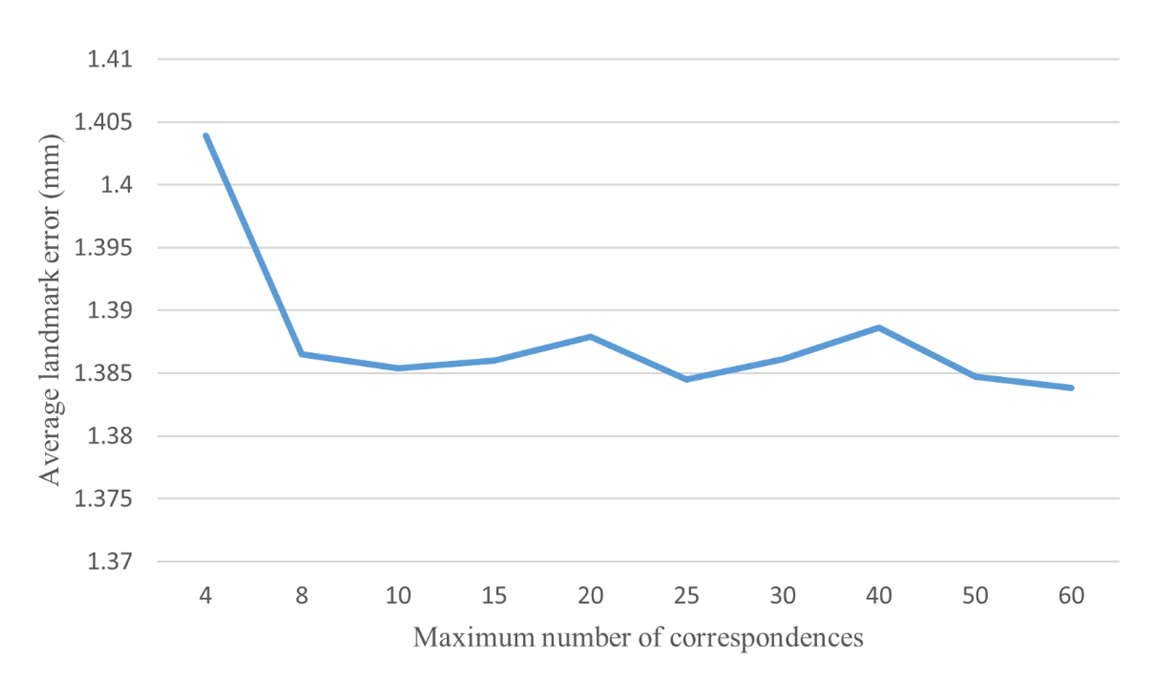

Supplement: S3 Fig — (TIF) [file pone.0207895.s003.tif]

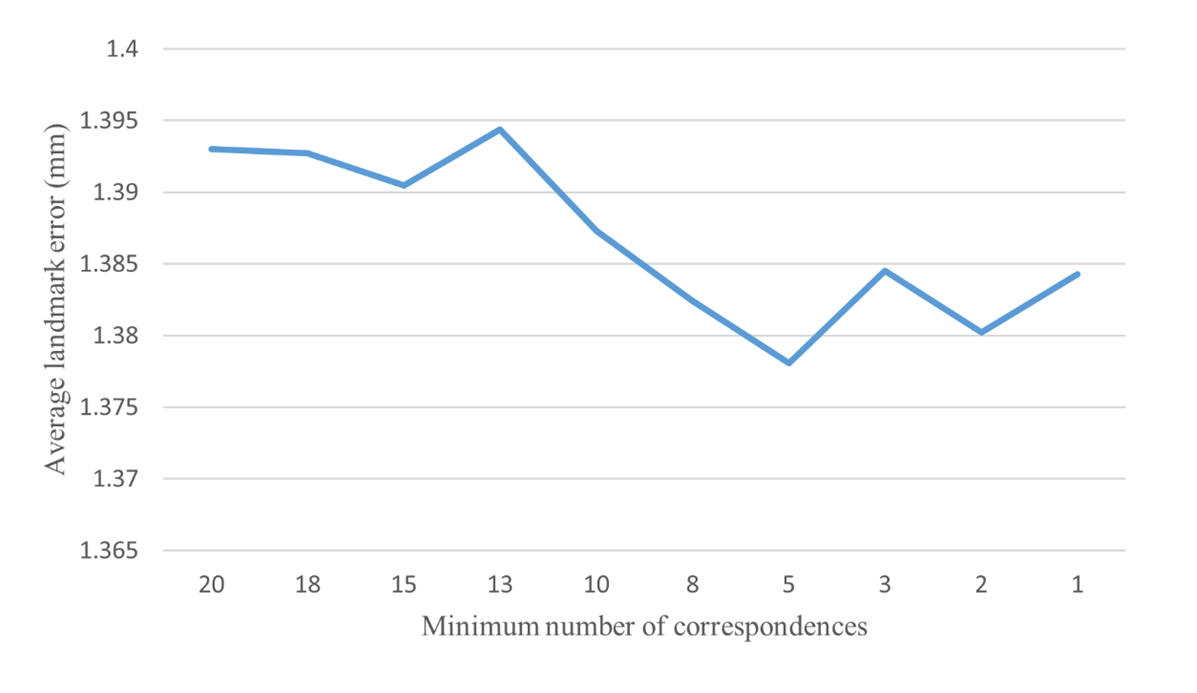

Supplement: S4 Fig — (TIF) [file pone.0207895.s004.tif]

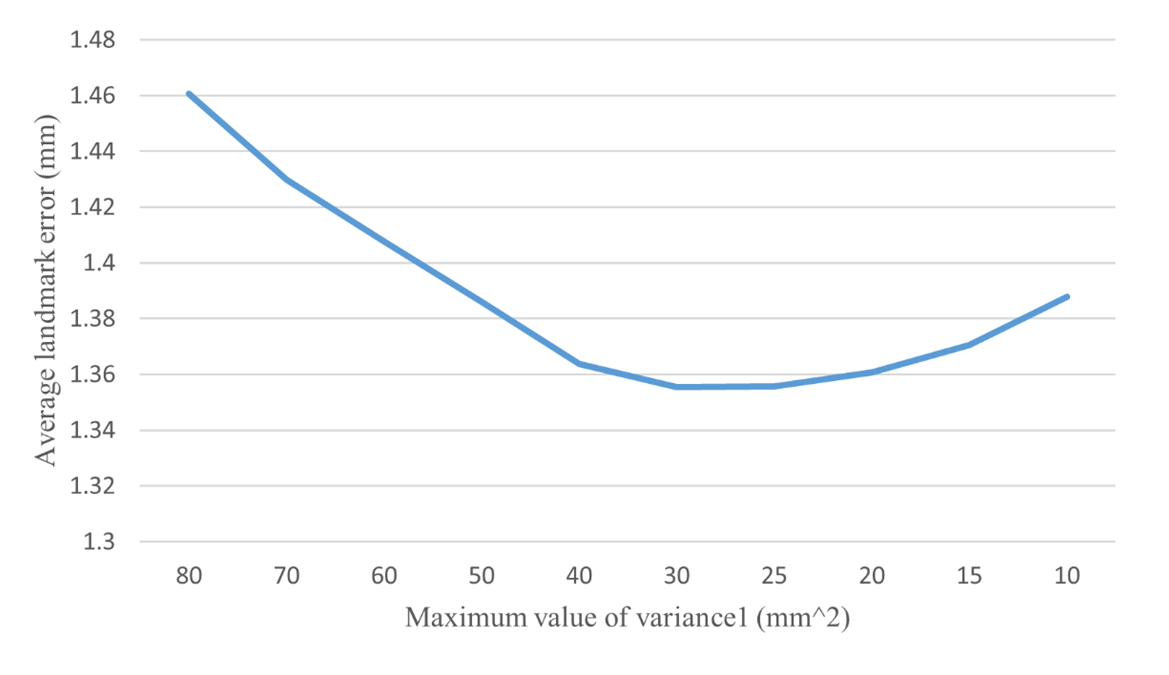

Supplement: S5 Fig — (TIF) [file pone.0207895.s005.tif]

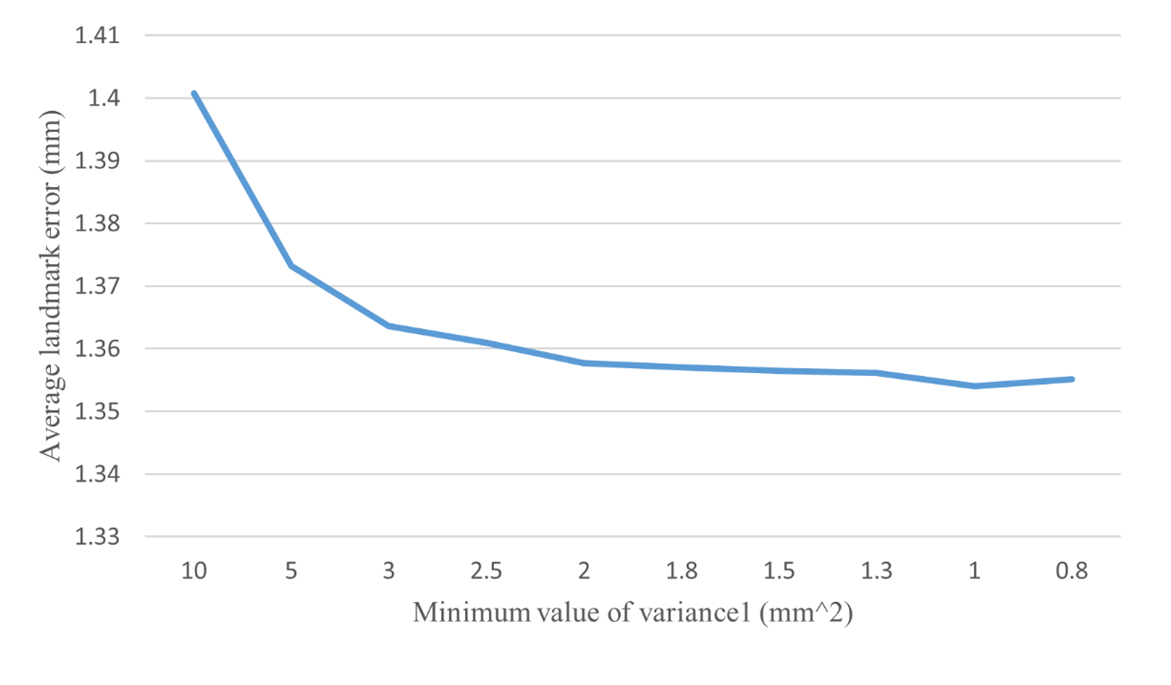

Supplement: S6 Fig — (TIF) [file pone.0207895.s006.tif]

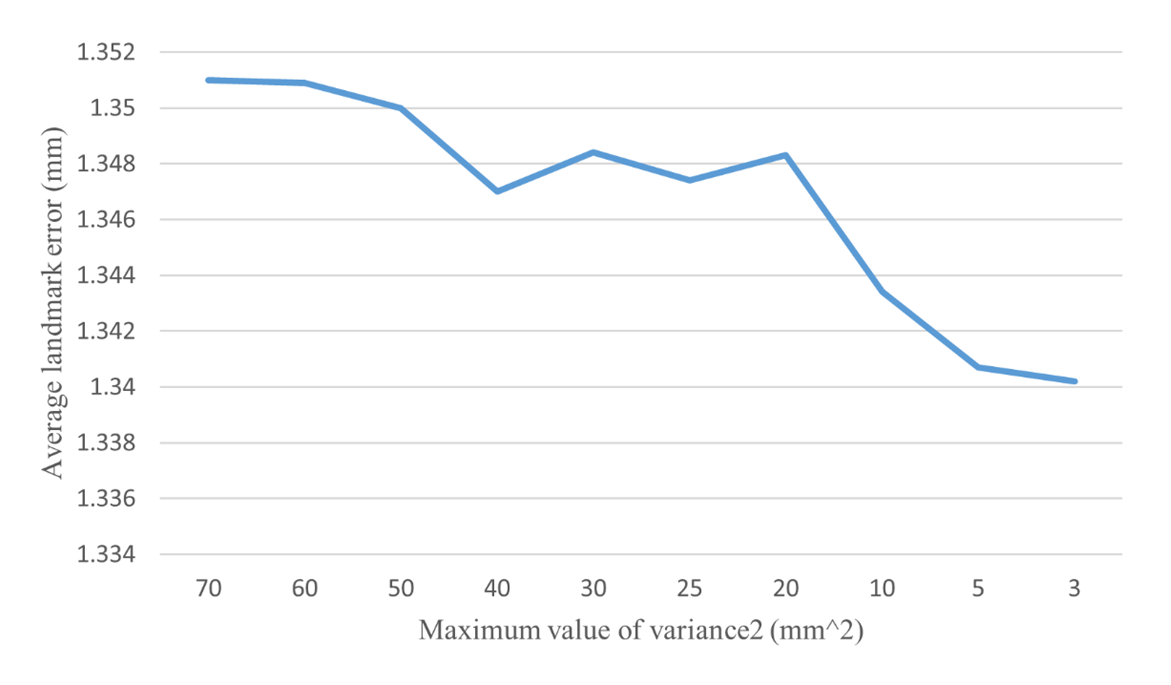

Supplement: S7 Fig — (TIF) [file pone.0207895.s007.tif]

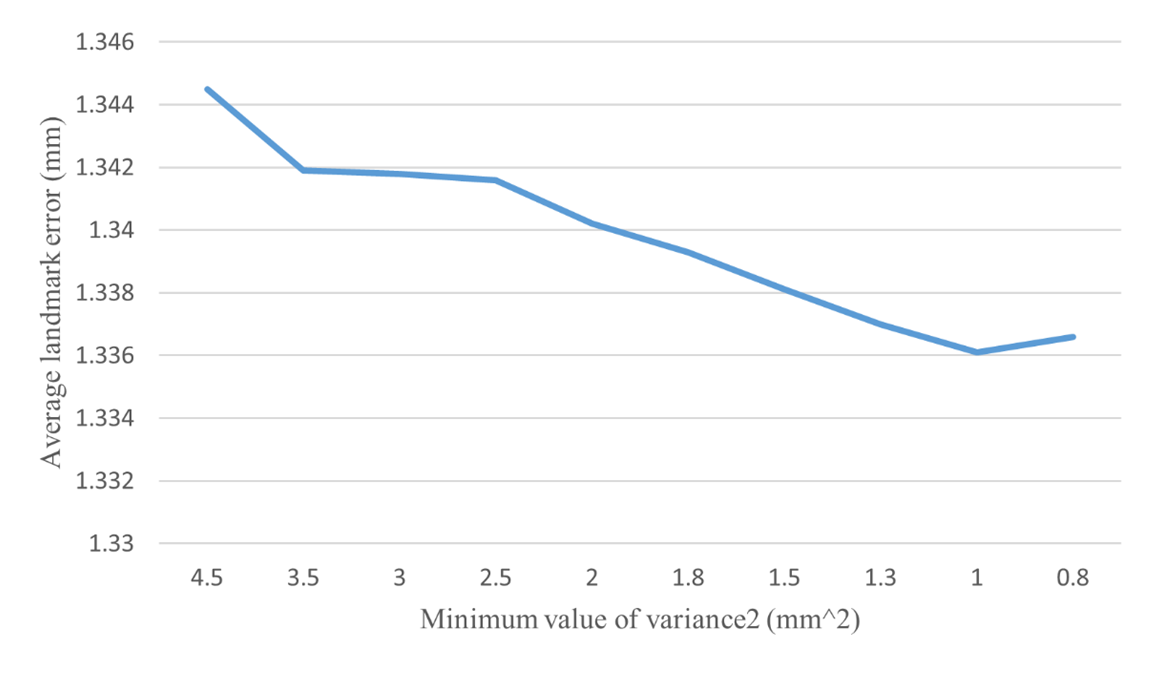

Supplement: S8 Fig — (TIF) [file pone.0207895.s008.tif]

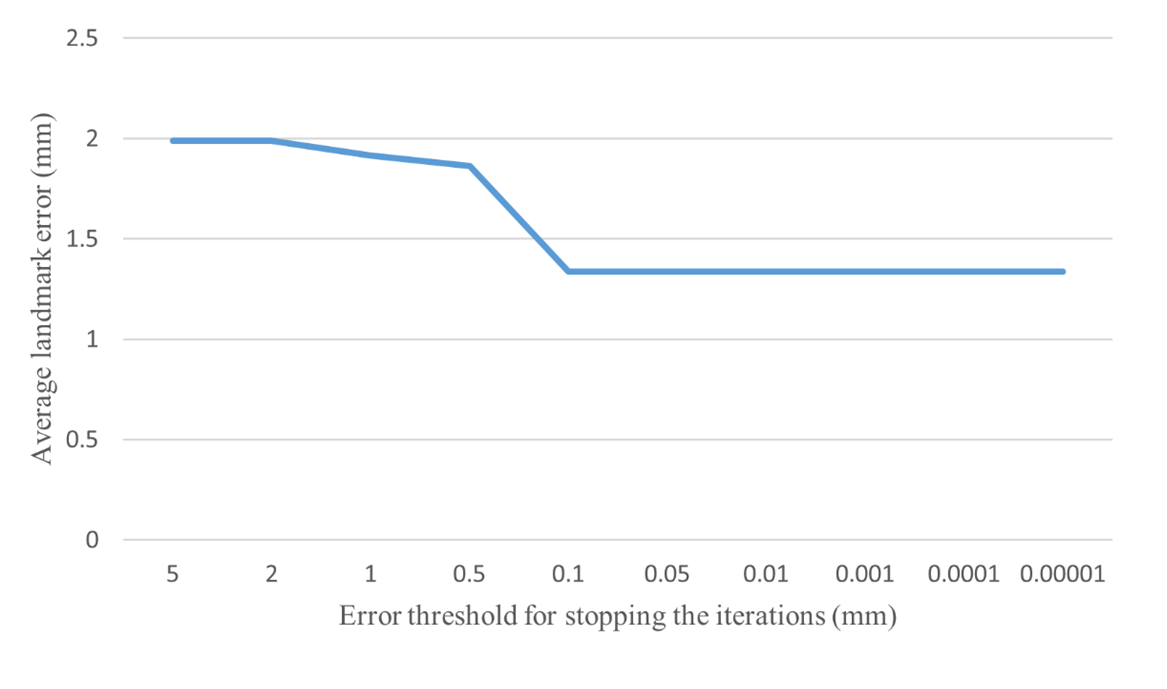

Supplement: S9 Fig — (TIF) [file pone.0207895.s009.tif]

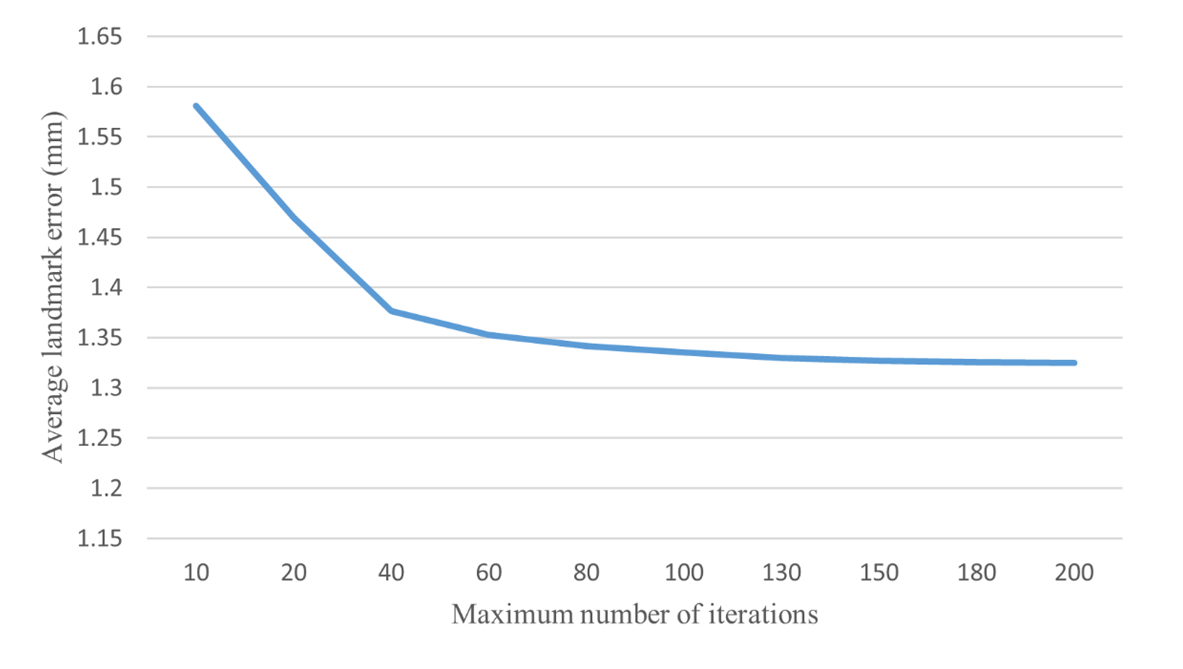

Supplement: S10 Fig — (TIF) [file pone.0207895.s010.tif]

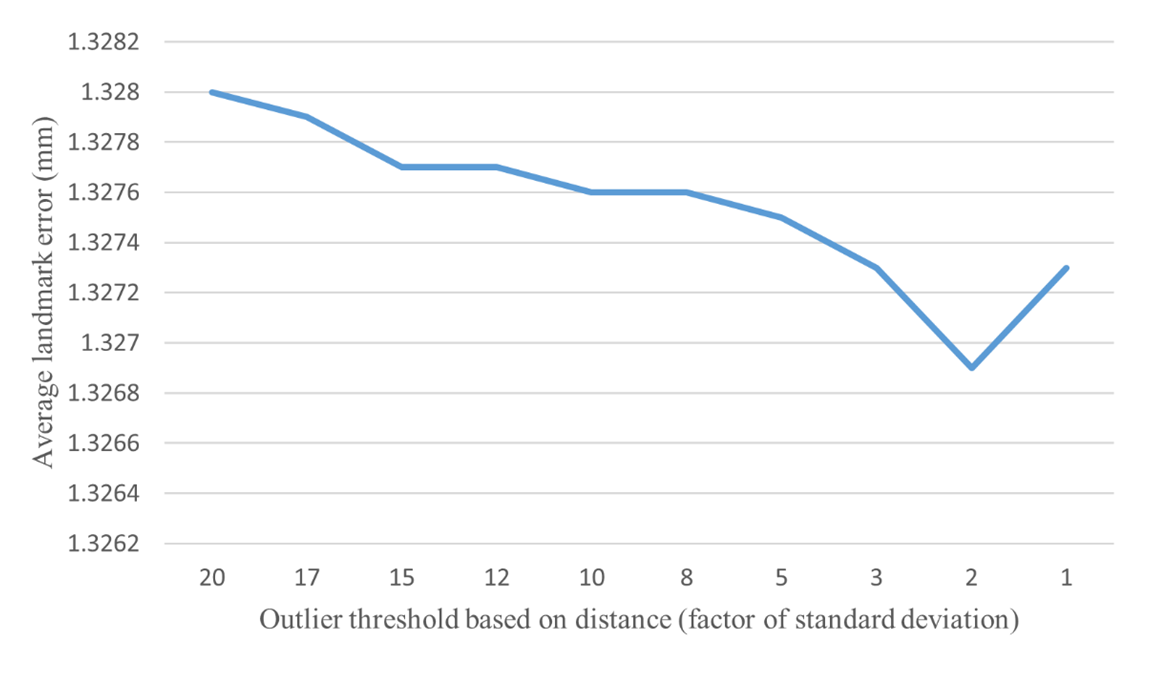

Supplement: S11 Fig — (TIF) [file pone.0207895.s011.tif]

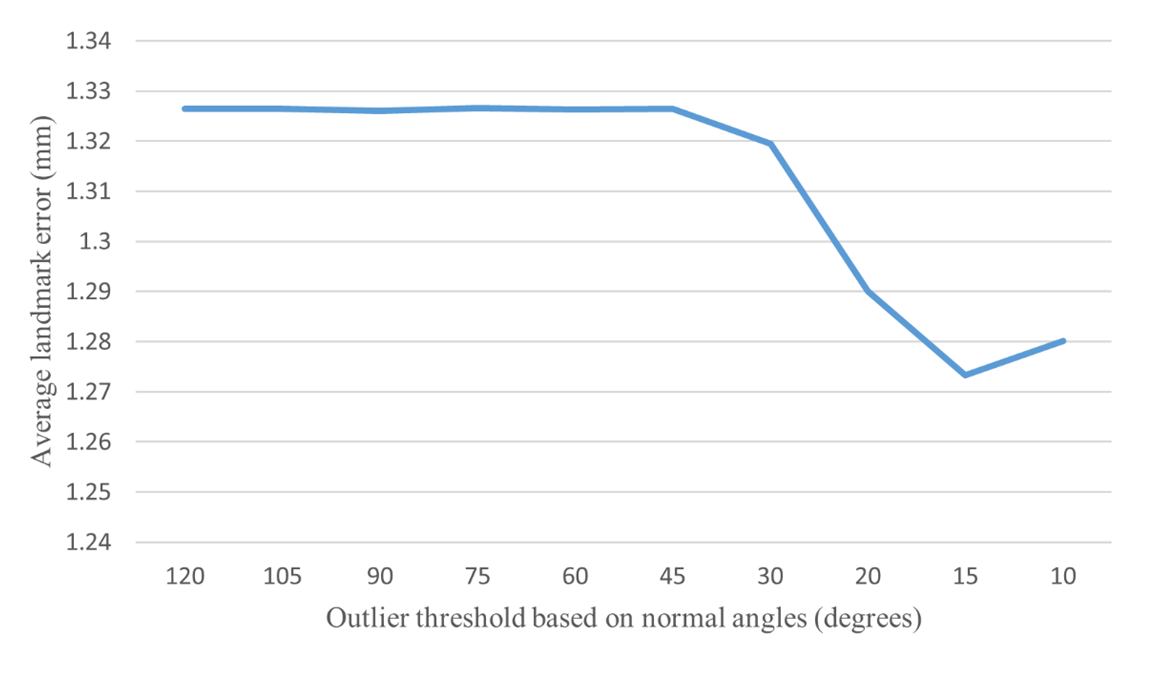

Supplement: S12 Fig — (TIF) [file pone.0207895.s012.tif]

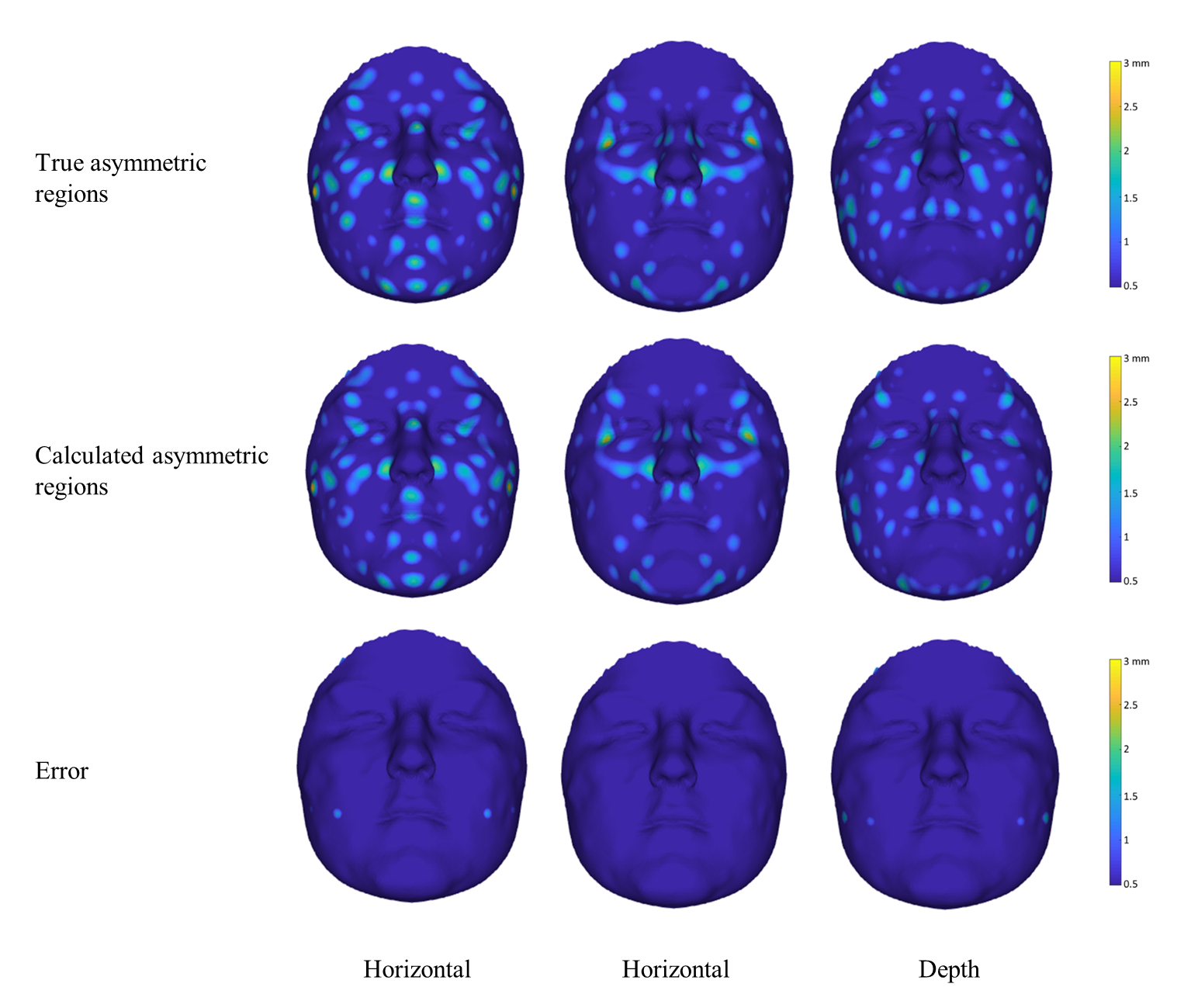

Supplement: S13 Fig — (TIF) [file pone.0207895.s013.tif]
